# Clinical profile of individuals with bisphosphonate-related osteonecrosis of the jaw: an integrative review

**DOI:** 10.1590/1516-3180.2019.0352.R2.15052020

**Published:** 2020-07-27

**Authors:** Aloizio Premoli Maciel, Reyna Aguilar Quispe, Lázara Joyce Oliveira Martins, Rogério Jardim Caldas, Paulo Sérgio da Silva Santos

**Affiliations:** I DDS, MSc. Dentist and Doctoral Student, Department of Surgery, Stomatology, Pathology and Radiology, Faculdade de Odontologia da Universidade de São Paulo (FOUSP), Bauru (SP), Brazil.; II DDS, MSc. Dentist and Doctoral Student, Department of Surgery, Stomatology, Pathology and Radiology, Faculdade de Odontologia da Universidade de São Paulo (FOUSP), Bauru (SP), Brazil.; III DDS, MSc. Dentist and Assistant Professor, Department of Radiology, Clinical School of Dentistry, Universidade de Rio Verde (UniRV), Rio Verde (GO), Brazil.; IV DDS, MSc, PhD. Dentist and Head, Department of Dentistry, Hospital Mário Kroeff - Hospital de Câncer, Rio de Janeiro (RJ), Brazil.; V DDS, MSc, PhD. Dentist and Associate Professor, Department of Surgery, Stomatology, Pathology and Radiology, Faculdade de Odontologia da Universidade de São Paulo (FOUSP), Bauru, São Paulo, Brazil.

**Keywords:** Osteonecrosis, Jaw, Diphosphonates, Bisphosphonate-associated osteonecrosis of the jaw, Dental care, Angiogenesis inhibitors, Osteonecrosis of the jaw, Medication-related osteonecrosis of the jaw, Neoplasm metastasis therapy, Diagnosis and management of osteonecrosis of the jaw, Drug-induced osteonecrosis of the jaw

## Abstract

**BACKGROUND:**

Bisphosphonate-related osteonecrosis of the jaw (BRONJ) is still the most prevalent type of osteonecrosis with clinical relevance. In Brazil, bisphosphonate use is high but there is a lack of epidemiological studies on BRONJ.

**OBJECTIVE:**

To determine the clinical profile of BRONJ in a Brazilian population through an integrative review.

**DESIGN AND SETTING:**

Integrative review of BRONJ in a Brazilian population.

**METHODS:**

Cases and clinical research on Brazilians with BRONJ between 2010 and 2019, indexed in PubMed/MEDLINE, Scopus, Web of Science and LILACS were reviewed. Age, sex, type and time of bisphosphonate intake, administration route, related diseases, region of the BRONJ, diagnostic criteria, staging, triggering factor and type of treatment were analyzed.

**RESULTS:**

Fifteen articles on 128 subjects were included. Most patients were women (82.03%); the mean age was 63 years. Intravenous zoledronic acid was mostly used (62.50%), for breast cancer treatment (46.87%). The main localization of BRONJ was the mandible (54.68%), associated mainly with tooth extractions (45.98%). The diagnostic criteria were clinical (100%) and radiographic (89.06%), mostly in stage II (68.08%). The surgical treatments were sequestrectomy (37.50%) and platelet-rich plasma (PRP) (36.71%). Microbial control was done using chlorhexidine (93.75%) and infection control using clindamycin (53.90%).

**CONCLUSIONS:**

BRONJ had higher prevalence in Brazilian women receiving treatment for breast cancer and osteoporosis. The mandible was the region most affected with a moderate stage of BRONJ, particularly when there were histories of tooth extraction and peri-implant surgery. Sequestrectomy with additional drugs and surgical therapy was the treatment most accomplished.

## INTRODUCTION

Bisphosphonates (BPs) are drugs with oncological indication that have been used since 1960.^1^ They are currently indicated as therapy for multiple myeloma, malignant hypercalcemia, prevention of bone metastases and pathological fractures.^2^ BPs may also be prescribed for other diseases such as rheumatoid arthritis, osteoporosis and osteopenia.^3^^,^^4^^,^^5^

The mechanism of action of BPs consists of decreasing local vascular support and regulating bone metabolism, thereby reducing the action of osteoclasts and decreasing angiogenesis. Therefore, bone remodeling and deposition of physiological bone matrix are also affected.^6^^,^^7^ These effects on bone metabolism associated with local triggering factors, such as infection and tissue inflammation in the mouth, are named bisphosphonate-related osteonecrosis of the jaw (BRONJ).^8^ Among the main triggering factors of BRONJ are the following: exodontia, peri-implant surgery and traumas in the buccal mucosa.^1^^,^^9^^,^^10^^,^^11^ The clinical characteristics of BRONJ can include asymptomatic manifestations, severe pain, presence of infections and bone exposure.^6^

In 2014, there was a change in the nomenclature for this disease, to take into account its relationship with other drugs. The names currently used follow the pattern [medication]-related osteonecrosis of the jaw. This relates to the use of anti-resorptive and antiangiogenic medications,^8^^,^^12^^,^^13^^,^^14^ and more recent studies also mention the use of tyrosine kinase-inhibitor drugs and mammalian-target inhibitors of rapamycin.^8^^,^^9^^,^^10^^,^^11^^,^^12^^,^^13^^,^^14^

Although, as mentioned before, other medications relating to maxillary osteonecrosis do exist, BPs are still the most relevant drugs in relation to osteonecrosis of the jaws.^8^ The Brazilian population has high rates of breast and prostate cancer,^15^ and for this reason, BPs are highly indicated, which exposes these individuals to the risk of BRONJ.

The clinical profile of BRONJ and treatment protocols can vary according to specific demographic factors. Therefore, there is a need for population-specific studies. However, there are no studies in the literature reporting on the features of BRONJ and its treatment in Brazil.

## OBJECTIVE

This integrative review aimed to determine the clinical profile of osteonecrosis of the jaw exclusively associated with bisphosphonate therapy in the Brazilian population.

## METHODS

The guiding question of this review was: What are the clinical features of BRONJ in the Brazilian population that determine its clinical profile?

The inclusion criterion for this integrative review was that publications relating to Brazilian individuals would be included: these could include case reports, case series and clinical studies. Over the last decade, the nomenclature, staging and treatment method for BRONJ have undergone changes. This review considered articles either in English or in Portuguese that were published between January 2010 and April 2019. Articles that comprised review of the literature, laboratory analyses, letters to the editor, studies conducted on animal studies and research that did not involve Brazilians were excluded.

The variables selected were the following: age, sex, type of bisphosphonate used, duration of use of bisphosphonates until disease manifestation, route of administration, underlying disease that led to indication for drug use, oral region affected by BRONJ, clinical criteria for diagnosis of BRONJ, clinical staging according to the American Association of Oral and Maxillofacial Surgeons (AAOMS),^8^ local triggering factor and type of treatment. When data on any of these variables were absent, the study was not included in this review.

Four online databases were searched for articles: PubMed, Scopus, Web of Science and LILACS. PubMed, Scopus and Web of Science are international databases that have a search filter for the nationality of the articles, and this was used after the initial search. LILACS is a Latin American database with descriptors in the English and Portuguese language. We used the country identification tool for the Scopus, Web of Science and LILACS, and for PubMed we add the descriptor “Brazil”. The descriptors entered in the databases are described below.

1. “osteonecrosis” and “bisphosphonate” and “Brazil” for PubMed.
2. “osteonecrosis” and “bisphosphonate” for Web of Science and Scopus
3. “osteonecrosis” and “bisphosphonate” and “bisphosphonate or “diphosphonate” and “osteonecrosis” for Lilacs.

**Figure 1.**
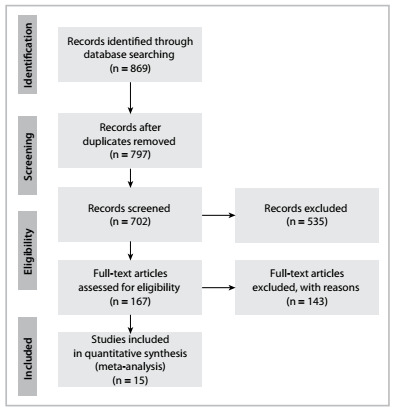
Flow diagram of the studies included in the integrative review

The selection of the articles that were assessed in full for the analysis on each of the variables of this review is described in Figure 1.

## RESULTS

Fifteen studies were included (Figure 1).^4^^,^^5^^,^^9^^,^^16^^,^^17^^,^^18^^,^^19^^,^^20^^,^^21^^,^^22^^,^^23^^,^^24^^,^^25^^,^^26^ A total of 141 different areas of BRONJ were reported in 128 individuals, of whom 105 (82.03%) were female^4^^,^^9^^,^^10^^,^^12^^,^^21^^,^^16^^,^^17^^,^^18^^,^^19^^,^^20^^,^^21^^,^^24^^,^^25^ and 23 (17.97%) were male.^5^^,^^9^^,^^10^^,^^16^^,^^17^^,^^18^^,^^19^^,^^25^ The subjects’ minimum age was 38 years and maximum age was 90 years, with a mean of 63.34 ± 34.87 years.

The commonest diagnoses relating to BRONJ were: breast cancer, in 60 individuals^9^^,^^10^^,^^12^^,^^16^^,^^17^^,^^19^^,^^21^ (46.87%); osteoporosis,^10^^,^^17^^,^^18^^,^^19^^,^^23^^,^^24^^,^^25^^,^^26^ in 25 (19.53%); multiple myeloma,^5^^,^^9^^,^^16^^,^^17^^,^^20^ in 16 (12.50%); and prostate cancer,^9^^,^^10^^,^^16^^,^^18^^,^^19^ in 16 (12.50%) (Table 1).^4^^,^^5^^,^^9^^,^^10^^,^^12^^,^^16^^,^^17^^,^^18^^,^^19^^,^^20^^,^^21^^,^^23^^,^^24^^,^^25^^.^^26^

**Table 1.** Reports on Brazilian individuals with bisphosphonate-related osteonecrosis of the jaw

Authors	Title	Type of bisphosphonate	No. of individuals	Gender	Age	Duration of bisphosphonate intake (months)	Related diseases
Antonini et al.^21^	Management of osteonecrosis of the jaws in patients with history of bisphosphonates therapy.	ZOE (1/1)	1	Female	72	36	BC
Conte-Neto et al.^4^	Oral bisphosphonate-related osteonecrosis of the jaws in rheumatoid arthritis patients: a critical discussion and two case reports.	ALE (2/2)	2	Female (2/2)	58-68	84-96	RA (2/2)
Curi et al.^9^	Bisphosphonate-related osteonecrosis of the jaws--an initial case series report of treatment combining partial bone resection and autologous platelet-rich plasma.	ZOE (21/25) PAM (4/25)	25	Female (20/25) Male (5/25)	42-85	12 - 84	BC (14/25) MM (7/25) PC (4/25)
Martins et al.^16^	Association of laser phototherapy with PRP improves healing of bisphosphonate-related osteonecrosis of the jaws in cancer patients: a preliminary study.	ZOE (18/22) PAM (4/22)	22	Female (16/22) Male (6/22)	42-90	12- 48	BC (13/22) PC (6/22) MM (2/22) LC (1/22)
Farias et al.^17^	Clinical and image findings in bisphosphonate-related osteonecrosis of the jaws.	ZOE (5/7) ALE (2/7)	7	Female (6/7) Male (1/7)	42-76	36 - 156	BC (2/7) OP (2/7) MM (1/7) LVC (1/7) GCC (1/7)
Rabelo et al.^18^	Bisphosphonate-Related Osteonecrosis of the Jaws and Its Array of Manifestations.	ZOE (4/8) ALE (4/8)	8	Female (6/8) Male (2/8)	53-76	5 - 72	OP (4/8) BC (2/8) PC (1/8) PD (1/8)
Mathias Duarte et al.^19^	Bisphosphonate-related osteonecrosis of the jaws: analysis of a case series at a dental school.	ZOE (7/13) PAM (2/13) ZOE + PAM (1/13) ALE (3/13)	13	Female (12/13) Male (1/13)	48-84	36	BC (9/13) OP (3/13) PC (1/13)
Zanata et al.^5^	Bisphosphonate-related osteonecrosis of the jaw in patient affected by multiple myeloma: A case report.	PAM (1/1)	1	Male	55	48	MM
Lopes et al.^10^	Surgical Therapy for Bisphosphonate-Related Osteonecrosis of the Jaw: Six-Year Experience of a Single Institution.	ZOE (22/33) ZOE + PAM (5/33) PAM (3/33) ALE (2/33) ZOE + ALE (1/33)	33	Female (25/33) Male (8/33)	39-83	26 - 120	BC (18/33) MM (4/33) PC (4/33) LC (4/33) OP (2/33) KC (1/33)
Heggendorn et al.^20^	Bisphosphonate-related osteonecrosis of the jaws: Report of a case using conservative protocol.	ZOE (1/1)	1	Female	52	22	MM
Maluf et al.^12^	Surgery Combined with LPRF in Denosumab Osteonecrosis of the Jaw: Case Report.	ZOE (1/1)	1	Female	79	36	BC
Momesso et al.^23^	Successful use of lower-level laser therapy in the treatment of medication-related osteonecrosis of the jaw.	ALE (1/1)	1	Female	65	60	OP
de Oliveira Ruellas et al.^24^	Managing bisphosphonate‐related osteonecrosis of the jaws with xenografts: a case report.	ALE (1/1)	1	Female	69	120	OP
Fernando de Almeida Mourão et al.^25^	The use of Platelet-rich Fibrin in the management of medication-related osteonecrosis of the jaw: A case series.	ALE (11/11)	11	Female (9/11) Male (2/11)	38-84	36-84	OP
Santos et al.^26^	Extensive osteonecrosis of the maxilla caused by bisphosphonates: Report of a rare case.	ZOE + ALE (1/1)	1	Female	52	144	OP
Total		ZOE 80 (62.50%) ALE 26 (20.31%) PAM 14 (10.93%) ZOE + PAM 6 (4.68%) ZOE + ALE 2 (1.56%)	128 (100%)	Female 105 (82.03%) Male 23 (17.97%)	63.34 (mean)	45 months (mean)	BC 60 (46.87%) OP 25 (19.53%) MM 16 (12.50%) PC 16 (12.50%) LC 5 (3.90%) RA 2 (1.56%) KC 1 (0.78%) PD 1 (0.78%) GCC 1 (0.78%) LVC 1 (0.78%)

ZOE = zoledronate; PAM = pamidronate; ALE = alendronate; BC = breast cancer; MM = multiple myeloma; PC = prostate cancer; OP = osteoporosis, LC = lung cancer; RA = rheumatoid arthritis; KC = kidney cancer; PD = Paget’s disease; GCCA = giant-cell cancer; LVC = liver cancer.

The types of BPs most related to BRONJ were: zoledronic acid ^9^^,^^10^^,^^12^^,^^16^^,^^17^^,^^18^^,^^19^^,^^20^^,^^21^^,^^26^ (ZOE), in a dose of 4 mg intravenously, in 80 cases (62.50%); and alendronate^4^^,^^10^^,^^17^^,^^18^^,^^19^^,^^23^^,^^24^^,^^25^^,^^26^ (ALE) in a dose of between 70 and 90 mg orally, in 26 cases (20.31%). However, other BPs such as pamidronate^5^^,^^9^^,^^10^^,^^16^^,^^19^ (PAM) (10.93%), an association of ZOE and PAM^10^^,^^19^^,^^26^ in six cases (4.68%) and an association of ZOE and ALE^10^^,^^26^ in two cases (1.56%) were also used. The duration of BP use until a manifestation of BRONJ ranged from 5 to 15 months, with a mean of 45.8 ± 39.7 months.^4^^,^^5^^,^^9^^,^^16^^,^^17^^,^^18^^,^^19^^,^^20^^,^^21^^,^^22^^,^^23^^,^^24^^,^^25^^,^^26^

Regarding the location of the BRONJ, the mandible was the most affected by BRONJ,^4^^,^^9^^,^^10^^,^^16^^,^^17^^,^^18^^,^^19^^,^^20^^,^^25^ in 70 cases (54.68%), followed by the maxilla^9^^,^^10^^,^^12^^,^^16^^,^^17^^,^^18^^,^^19^^,^^21^^,^^23^^,^^24^^,^^25^^,^^26^ in 52 cases (40.62%). Less frequent manifestations were in both jaws,^16^^,^^17^^,^^19^ the palatine torus^10^ and the mylohyoid region^17^ (Table 2).^4^^,^^5^^,^^9^^,^^10^^,^^12^^,^^16^^,^^17^^,^^18^^,^^19^^,^^20^^,^^21^^,^^23^^,^^24^^,^^25^^,^^26^

**Table 2.** Clinical features of bisphosphonate-related osteonecrosis of the jaw (BRONJ)

Authors	Title	Region of BRONJ	No. of cases of BRONJ	Triggering factor	Diagnostic criteria	**AAOMS classification (stage)**	Type of treatment
Antonini et al.^21^	Management of osteonecrosis of the jaws in patients with history of bisphosphonates therapy.	Post. right reg. maxilla	1	Spontaneous	CLN, TOMO and HPT	III (1/1)	HBO therapy, RES, PRP, CFLX, CHX, DH
Conte-Neto et al.^4^	Oral bisphosphonate-related osteonecrosis of the jaws in rheumatoid arthritis patients: a critical discussion and two case reports.	Right reg. mandible; left reg. mandible	2	Trauma; periodontitis	CLN, TOMO, and HPT; CLN, RAD and TOMO	II (2/2)	AT Clin. CHX, IR, SEQ, AdDEB; CHX, AT Clav. EXT, AdDEB.
Curi et al.^9^	Bisphosphonate-related osteonecrosis of the jaws--an initial case series report of treatment combining partial bone resection and autologous platelet-rich plasma.	Mandible (8/25); maxilla (7/25)	25	Extraction (14/25); prosthetic trauma (7/25); implant (2/25); spontaneous (2/25)	CLN and RAD	I (3/25) II (15/25) III (7/25)	CHX, AT; AT Clin, RES, AdDEB, PRP; EXT AdDEB, PRP
Martins et al.^16^	Association of laser phototherapy with PRP improves healing of bisphosphonate-related osteonecrosis of the jaws in cancer patients: a preliminary study.	Mandible (17/22); maxilla (3/22); both jaws (2/22)	22	Extraction (12/22); prosthetic trauma (3/22); spontaneous (2/22); periodontitis (1/22)	CLN, RAD and TOMO	I (9/22) II (10/22) III (3/22)	CHX + AT (3/22); RES + AdDEB (5/22); PRP + LLT (14/22)
Farias et al.^17^	Clinical and image findings in bisphosphonate-related osteonecrosis of the jaws.	Post. bilateral reg. mandible (2/7); right myeloid reg. (1/7); both post. reg. jaws (1/7); both post. left reg. jaws (1/7); ant. reg. maxilla (2/7)	7	Extraction (4/7); Extraction + Implant. (1/7); spontaneous (2/7)	CLN and RAD	0 (2/7) I (1/7) II (4/7)	DEBL + AT Amox + CHX
Rabelo et al.^18^	Bisphosphonate-Related Osteonecrosis of the Jaws and Its Array of Manifestations.	Post. reg. mandible (3/8); post. reg. maxilla (2/8); ant. reg. mandible (1/8); ant. reg. maxilla. (1/8); ant. and post. reg. maxilla (1/8)	8	Extraction (6/8); spontaneous (2/8)	CLN and RAD	0 (1/8) I (3/8) II (1/8) III (3/8)	AT + DEBL; AT + SURG; AS + AT; AT + AS + DEBL/ AT + DEBL
Mathias Duarte et al.^19^	Bisphosphonate-related osteonecrosis of the jaws: analysis of a case series at a dental school.	Mandible (8/13); maxilla (4/13); both jaws (1/13)	13	Extraction (7/13); implant (2/13); periodontitis (2/13); spontaneous (2/13)	CLN, RAD and TOMO	II (13)	CHX, AT Clin. (3/13); RES (4/13); PRP (6/13)
Zanata et al.^5^	Bisphosphonate-related osteonecrosis of the jaw in patient affected by multiple myeloma: A case report.	Post. reg. mandible	1	Extraction	CLN, RAD and TOMO	II (1/1)	OH + CHX +SEQ + AdDEB
Lopes et al.^10^	Surgical Therapy for Bisphosphonate-Related Osteonecrosis of the Jaw: Six-Year Experience of a Single Institution.	Post. reg. mandible. (19/46); post. reg. maxilla (16/46); Ant. reg. mandible (5/46); Ant. reg. maxilla. (3/46); mandibular torus (2/46); both jaws (1/46)	46	Extraction (16/46); periodontitis + periimplantitis (9/46); prosthetic trauma (8/46); spontaneous (8/46); implant (3/46); palatine torus trauma (2/46)	CLN and RAD	II (37/46) III (9/46)	Ad DEB; SEQ; AT Clin + AT + CHX
Heggendorn et al.^20^	Bisphosphonate-related osteonecrosis of the jaws: Report of a case using conservative protocol.	Post. left. reg. mandible	1	Prosthetic trauma	CLN and RAD	II (1/1)	CHX + LLLT
Maluf et al.^12^	Surgery Combined with LPRF in Denosumab Osteonecrosis of the Jaw: Case Report.	Ant. reg. maxilla	1	Extraction	CLN and TOMO	III (1/1)	DEB, LPRF, CHX, Amox + CLV, MTZ, DH
Momesso et al.^23^	Successful use of lower-level laser therapy in the treatment of medication-related osteonecrosis of the jaw.	Post. right reg. maxilla.	1	Implant	CLN and RAD	II (1/1)	LLLT + CHX + Clin
de Oliveira Ruellas et al.^24^	Managing bisphosphonate‐related osteonecrosis of the jaws with xenografts: a case report.	Post. right reg. maxilla.	1	Odontogenic infection	CLN and TOMO	III (1/1)	Ress, EXT, graft, AdDEB, CFLX, MTZ, CHX
Fernando de Almeida Barros Mourão et al.^25^	The use of Platelet-rich Fibrin in the management of medication-related osteonecrosis of the jaw: A case series.	Ant. reg. maxilla (1/11); post. right reg. maxilla (3/11); post. left reg mandible (3/11); post. right reg. mandible (4/11)	11	Implant (10/11); extraction (1/11)	CLN, RAD and TOMO	II (11/11)	RES, PRP, AdDEB, CHX
Santos et al.^26^ 2019	Extensive osteonecrosis of the maxilla caused by bisphosphonates: Report of a rare case.	Ant. and post. bilateral reg. maxilla (1/1).	1	Extraction	CLN and TOMO	III (1/1)	CHX, Amox + CLV, RES, AdDEB
Total		Post. reg. mandible 33 (25.78%); post. reg. maxilla 24 (18.75%); ant. reg. maxilla 7 (5.46%); ant. reg. mandible 6 (4.68%); post. reg. both jaws 2 (1.56%); palatine torus 2 (1.56%); myeloid reg. 1 (0.78%); post. and ant. reg. both jaws 1 (0.78%)	141 (100%)	Extraction 63 (45.98%); implant 19 (13.86%); prosthetic trauma 19 (13.86%); spontaneous 19 (13.86%); periodontitis 13 (10.65%); palatine torus trauma 2 (1.45%); trauma 1 (0.73%); odontogenic infection 1 (0.72%) 137 (100%)	CLN 128 (100%); RAD 114 (89.06%); TOMO 52 (40.62%); HPT 3 (2.34%) 128 (100%)	Stage 0: 3 (2.12%); stage I: 16 (11.34%); stage II: 96 (68.08%); Stage III: 26 (18.43 %) 141 (100%)	CHX 120 (93.75%); Clin 69 (53.90%); AT 54 (42.18%); SEQ + adDEB 48 (37.50%); PRP 47 (36.71%); EXT + adDEB 26 (20.31%); LLLT 16 (12.50%); RES + AdDEB 13 (10.15%); Amox 4 (3.12%); AD 3 (2.34%); DH 2 (1.56%); IR 1 (0.78%); LPRF 1 (0.78%); HBO 1 (0.78%) 128 (100%)

AT = other antibiotics; CHX = chlorhexidine; Ext = extraction; AdDEB = additional debridement; SEQ = sequestrectomy; RES = resection; IR = implant removal; CLN = clinical; RAD = radiographic; TOMO = tomographic; HPT = histopathologically; DEBL= local debridement; Reg. = region; PRP = platelet-rich plasma; LLLT = low-level laser therapy; Post. = posterior; Ant = anterior; OH = oral hygiene guidance; Clin = clindamycin; AD = abscess drainage; Amox = amoxicillin; MTZ = metronidazole; CFLX = cefalexin; DH = drug holiday; HBO = hyperbaric oxygen therapy; LPRF = leukocyte-platelet-rich fibrin.

The main diagnostic criterion was clinical evaluation of bone exposure,^4^^,^^5^^,^^9^^,^^16^^,^^17^^,^^18^^,^^19^^,^^20^^,^^21^^,^^22^^,^^23^^,^^24^^,^^25^^,^^26^ which was found to be present in 100% of the cases. The most common complementary examinations were panoramic radiographs,^4^^,^^5^^,^^9^^,^^10^^,^^16^^,^^17^^,^^18^^,^^19^^,^^20^^,^^23^^,^^25^ in 114 cases (89.06%); and cone-bean computed tomography,^4^^,^^5^^,^^12^^,^^16^^,^^19^^,^^21^^,^^24^^,^^25^^,^^26^ in 52 cases (40.62%). In contrast, the histopathological evaluation^4^^,^^21^ was present only in three cases (2.34%) (Table 2).^4^^,^^5^^,^^9^^,^^10^^,^^12^^,^^16^^,^^17^^,^^18^^,^^19^^,^^20^^,^^21^^,^^23^^,^^24^^,^^25^^,^^26^

The local trigger factors for BRONJ were the following: tooth extraction,^5^^,^^9^^,^^10^^,^^12^^,^^16^^,^^17^^,^^18^^,^^19^^,^^25^^,^^26^ in 63 individuals (45.98%); implant placement,^9^^,^^17^^,^^19^^,^^23^^,^^25^ in 19 (13.86%); prosthetic trauma,^9^^,^^10^^,^^16^^,^^20^ in 19 (13.86%); and spontaneous manifestation,^9^^,^^10^^,^^16^^,^^17^^,^^18^^,^^19^^,^^21^ in 19 (13.86%). Other local factors are considered in Table 2.^4^^,^^5^^,^^9^^,^^10^^,^^12^^,^^16^^,^^17^^,^^18^^,^^19^^,^^20^^,^^21^^,^^23^^,^^24^^,^^25^^,^^26^

According to the AAOMS classification, three individuals (2.12%) presented stage 0^17^^,^^18^ of BRONJ; 16 cases (11.34%) had stage I;^9^^,^^16^^,^^17^^,^^18^ 96 cases (68.08%, i.e. the majority) presented stage II;^4^^,^^5^^,^^9^^,^^10^^,^^16^^,^^17^^,^^18^^,^^19^^,^^20^^,^^23^^,^^25^ and 26 cases (18.43%) had stage III^9^^,^^10^^,^^12^^,^^16^^,^^18^^,^^21^^,^^24^^,^^26^ (Table 2).^4^^,^^5^^,^^9^^,^^10^^,^^12^^,^^16^^,^^17^^,^^18^^,^^19^^,^^20^^,^^21^^,^^23^^,^^24^^,^^25^^,^^26^

There was a lack of detailed information about the types of treatment and management used in these cases reported from Brazilian populations. The treatment most reported was sequestrectomy,^4^^,^^5^^,^^10^ in 48 cases (37.50%); while platelet-rich plasma^9^^,^^16^^,^^19^^,^^21^^,^^25^ was used to complement surgery, in 47 cases (36.71%). Other treatments, such as bone resection with or without curettage,^9^^,^^16^^,^^19^^,^^21^^,^^24^^,^^25^^,^^26^ tooth extractions with debridement of the necrotic bone,^4^^,^^9^ debridement alone^12^^,^^17^^,^^18^ or even low power laser therapy, are described in Table 2.^4^^,^^5^^,^^9^^,^^10^^,^^12^^,^^16^^,^^17^^,^^18^^,^^19^^,^^20^^,^^21^^,^^23^^,^^24^^,^^25^^,^^26^

The topical medication most used for treatment of BRONJ was chlorhexidine solution,^4^^,^^5^^,^^9^^,^^16^^,^^17^^,^^19^^,^^20^^,^^21^^,^^22^^,^^23^^,^^24^^,^^25^^,^^26^ in 120 individuals (93.75%). The systemic medication used consisted of antibiotic therapy with clindamycin in 69 cases (53.90%) and amoxicillin in four cases (3.12%).^12^^,^^17^^,^^26^ Other antibiotics were also reported to have been used as part of BRONJ treatments but without any detailed description. Thus, for this reason, they were not included in this review.

## DISCUSSION

The cases of BRONJ in Brazil showed that 103 individuals (80.47%) received BPs intravenously as part of their cancer treatment. The other 25 (19.53%) received BPs orally as osteoporosis treatment. In North American populations, similar results were observed, i.e. BRONJ developed mainly in individuals who were undergoing oncological treatment intravenously.^27^^,^^28^ Use of this administration route increases the risk of BRONJ one hundredfold, compared with the oral route.^8^

The oral route for BPs is more used for controlling osteoporosis: not only in Brazil^4^^,^^5^^,^^9^^,^^16^^,^^17^^,^^18^^,^^19^^,^^20^^,^^21^^,^^22^^,^^23^^,^^24^^,^^25^^,^^26^ but also in the United States.^27^^,^^28^ Among BPs, ALE is the BP that is most associated with BRONJ. ^4^^,^^5^^,^^9^^,^^16^^,^^17^^,^^18^^,^^19^^,^^20^^,^^21^^,^^22^^,^^23^^,^^24^^,^^25^^,^^26^ In Europe, individuals with rheumatoid arthritis have higher incidence of BRONJ than individuals with osteoporosis, since ALE is the treatment of choice and its use is prolonged.^29^^,^^30^^,^^31^

In addition to the type of diagnosis of the disease and type of BP, surgical manipulation of the jaws^19^^,^^32^ accounts for 60% of the local trigger factors for developing BRONJ.^32^^,^^33^^,^^34^ Thus, in these Brazilian cases, the triggering factor most reported was tooth dental extraction (45.98%), especially in individuals using ZOE (62.50%). Tooth extraction was also reported to be the main local factor that triggered BRONJ in North American,^27^ European^31^^,^^34^^,^^35^^,^^36^^,^^37^^,^^38^^,^^39^ and Asian ^40^^,^^42^^,^^43^ populations.

Among the triggering factors for BRONJ, implant surgery still remains a matter of controversy in the literature.^8^ A systematic review found that there was no evidence to demonstrate safety in performing dentoalveolar surgical procedures such as placement of dental implants in individuals exposed to BPs. Therefore, such procedures should be considered to be local risk factors.^44^ In one Brazilian population, implant placement was considered to be the second most prevalent trigger factor (13.86%), with epidemiological values similar to those of a European population (13.50%).^35^ Placement of dental implants in individuals who are using BPs presents local and systemic risk factors for development of BRONJ, regardless of the route of BP administration. Hence, these patients should not be treated in a conventional manner. The imminent risk of BRONJ and the risk of failure of peri-implant treatment should be always considered in drawing up the treatment plan. Patients should always be made aware of their systemic and dental condition.

Among the jaw bones, the chance of developing BRONJ is twice as high in the mandible as in the maxilla.^8^^,^^32^^,^^42^^,^^45^ In the present review, we observed greater involvement of the mandible, i.e. similar to the findings in North American,^27^^,^^30^^,^^46^ European^31^^,^^34^^,^^35^^,^^37^^,^^38^^,^^39^^,^^47^^,^^48^ and Asian^40^^,^^41^^,^^42^^,^^43^ populations. Despite the lack of detailed information regarding the location of BRONJ that was seen in the present review, we found that the posterior region of the mandible was the most involved, which coincided with findings from the rest of the world’s population.^31^^,^^34^^,^^35^^,^^37^^,^^38^^,^^39^^,^^40^^,^^41^^,^^42^^,^^43^^,^^47^^,^^48^ Therefore, in all cases, bone manipulation must be done in a precise, fast and atraumatic manner.

The fact that women have been found to be more affected by BRONJ, both in Brazil and in the rest of the world,^27^^,^^28^^,^^29^^,^^30^^,^^31^^,^^34^^,^^36^^,^^37^^,^^38^^,^^39^^,^^40^^,^^41^^,^^42^^,^^43^^,^^47^^,^^48^^,^^49^^,^^50^ may be related to administration of BPs after the menopause and to high incidence of breast cancer and osteoporosis. In Brazil, under these two conditions, there is an indication for using BPs. In the present study, breast cancer and osteoporosis were the underlying diseases that led to the highest prevalence of BRONJ, and similar results were found in European populations.^36^^,^^38^ Although the prevalence of BRONJ has mainly been associated with occurrences of breast cancer in the United States, multiple myeloma is the second most prevalent disease related to this oral complication.^27^^,^^34^^,^^38^^,^^46^ The South Korean population is the only population in which BRONJ is more related to osteoporosis than to other underlying diseases.^42^^,^^43^

Therefore, it is important for medical specialists such as mastologists and/or gynecologists to be aware that patients who use BPs are at greater risk of developing BRONJ. One preventive measure could be to refer patients for dental assessment, before or during the first months of prescription of BPs, in order to eliminate some foci of infection that can expose these patients to the risk of developing BRONJ.

This integrative review identified that the majority of the Brazilian cases were diagnosed during stage 2 of BRONJ, and this is similar to findings from other countries.^30^^,^^31^^,^^36^^,^^37^^,^^38^^,^^41^^,^^42^^,^^43^^,^^46^^,^^48^ High prevalences of other stages are unusual, but when this occurs, it is usually stage 3, in which there is involvement of adjacent structures such as the maxillary sinus or occurrence of pathological fracture of the mandible.^35^^,^^47^^,^^48^^,^^49^ In the present review, stage 3 had the second highest prevalence, affecting 18.43% of this Brazilian population.

The diagnosis of BRONJ in these Brazilian cases was clinical in all of them. Since 2014, AAOMS has recommended the use of complementary imaging tests to finalize the staging and evaluate possible bone alterations that can precede BRONJ. Despite this recommendation for concomitant use of computed tomography (CT) as the most appropriate examination, the present review identified that only 40.62% of the cases were diagnosed by means of cone beam computed tomography (CBCT). This suggests not only that there is probably a lack of knowledge of indication of 3D imaging such as CBCT to perform better examinations, but also that there is a lack of local resources or else that these examinations have a high cost. The radiographic evaluation criterion was not included in this integrative review because of the lack of detailed information in the studies selected.

In addition, it is important to mention that, although AAOMS recommends that BRONJ should be diagnosed using clinical and imaging methods, we would emphasize that there is a need to make differential diagnoses in relation to other lesions with clinical signs of bone exposure, such as bone metastases and clinical manifestations of multiple myeloma in the jaw through histopathological analysis.^50^^,^^51^^,^^52^ Nonetheless, such lesions have been found to be very scarce, accounting for only 2.34%.

The etiology and progression of BRONJ are related to infection and inflammation.^8^ In these Brazilian cases, sequestrectomy, resection and curettage were used, almost always in association with chlorhexidine mouthwashes and antibiotic therapy when necessary. In this last case, clindamycin was the main antibiotic selected, while other antibiotics like amoxicillin, tetracycline and metronidazole were also used but less frequently. Some studies have reported that the penicillin group was the first choice among antibiotics in Europe,^34^^,^^35^^,^^47^ and clarithromycin in Asia.^40^

Among the types of treatment mentioned earlier, surgical treatment is widely used in different populations around the world.^29^^,^^34^^,^^41^^,^^46^^,^^47^^,^^53^ Regardless of the type of surgical approach used, debridement or sequestrectomy until accessing the bleeding bone is recommended for improving the chances of success in the treatment.^38^

The limitation of the present study was its inability to provide detailed information about the location of BRONJ, type and dose of medications, radiographic features, biopsy and follow-up because of the lack of detailed information in the studies selected. In addition, there were no randomized studies or investigations on BRONJ in Brazilian populations. For this reason, we suggest that such studies need to be conducted and need to provide detailed information, as mentioned earlier.

## CONCLUSION

The manifestation of BRONJ in this Brazilian population was greatest in the mandibles of younger females, with greater associations with breast cancer and osteoporosis. The major risk factor was previous exodontia, and BRONJ was diagnosed mainly in the intermediate staging (II). Surgical intervention was the treatment most commonly used among these Brazilian patients. This review identified greater use of chlorhexidine solution and prescription of clindamycin as the first-choice antibiotic therapy. PRP was the complementary therapy most used.
